# Downstream Imaging Studies Do Not Significantly Improve Outcome in Most Patients with Chest Pain Who Did Not Reach Their Target Heart Rate on a Stress ECHO Study

**DOI:** 10.3390/jcm12144832

**Published:** 2023-07-22

**Authors:** Nativ Henkin, Ifat Karilker, Sergio L. Kobal, Rachel Golan, Aryeh Shalev, Shaul Atar, Yaakov Henkin

**Affiliations:** 1 Department of Family Medicine, Clalit Health Services, Sharon-Shomron District, Kfar-Saba 4428164, Israel; 2 Clalit Health Services, Southern District, Dimona 8604113, Israel; 3 Department of Cardiology, Soroka University Medical Center, Beer-Sheva 8400101, Israel; 4 Faculty of Health Sciences, Ben-Gurion University of the Negev, Beer-Sheva 8410501, Israel; 5 Department of Epidemiology, Biostatistics and Community Health Sciences, Ben-Gurion University of the Negev, Beer-Sheva 8410501, Israel; 6 Department of Cardiology, Galilee Medical Center, Nahariya 2210001, Israel; 7 Azrieli Faculty of Medicine, Bar Ilan University, Safed 5290002, Israel

**Keywords:** stress echo, target heart rate, downstream imaging tests

## Abstract

Echocardiographic stress tests are often used to evaluate patients who complain of chest pain. However, some patients fail to reach the target heart rate required for the test to be conclusive (usually defined as 85% of the predicted maximal heart rate based on the patient’s age) and are often sent for additional imaging tests, such as myocardial perfusion imaging (MPI) or cardiac computed tomography angiography (CTA). Few studies have evaluated the effectiveness of these additional tests in patients who present with chest pain but did not meet the heart rate requirements for a stress test. The primary objective of the study was to evaluate the efficacy of additional imaging tests for patients who experience chest pain during daily activities but are unable to reach the target heart rate currently required for an echocardiographic stress test. The study group included 415 consecutive patients who underwent a stress echocardiogram, did not achieve their target heart rate, and did not demonstrate abnormal changes during the test. The control group consisted of 415 consecutive patients who did reach their target heart rate and demonstrated no signs of ischemia. Demographic and clinical data, medication use, imaging test results (MPI, CTA, and/or coronary catheterization) and documented cardiac events that occurred during 1 year of follow-up were obtained from the electronic medical records. Of the 415 patients in the study group, 73 (17.6%) were referred to another imaging test within 12 months. Of these 73 patients, 59 underwent MPI and 14 underwent cardiac CTA. In 12 of these patients (16.4%) the test was considered to be abnormal, but only 7 patients (1.7%) subsequently underwent a percutaneous intervention (PCI). In the control group, 28 (6.7%) patients were referred for another imaging test. Of these 28 patients, 14 underwent MPI and 14 underwent cardiac CTA. None of these tests were found to be abnormal, but two patients (0.5%) underwent a PCI (*p* = 0.2 between groups). There were no deaths during the study period and no patients underwent bypass surgery. The majority of the patients who underwent PCI had additional clinical risk factors (diabetes, hypertension, and/or known coronary artery disease), had taken a beta blocker within 24 h prior to the test, and/or did not reach a heart rate above 78% of their target heart rate. Our study suggests that in most patients with chest pain who do not show ischemic changes on a stress echocardiogram, additional imaging studies can be safely deferred, even if the required target heart rate was not reached. However, in patients with diabetes and/or known coronary disease, those who took a beta blocker 24 h prior to the test, or those who did not achieve a heart rate above 78% of the current target heart rate, additional imaging studies should be considered.

## 1. Introduction

Ischemic heart disease (IHD) is a leading cause of death globally. Although chest pain is the most common symptom of IHD, it can result from musculoskeletal, pulmonary, gastrointestinal, or psychiatric causes [1]. In cases where the cause of chest pain is unclear and there is an intermediate to high likelihood of cardiac ischemia, guidelines recommend referring the patient for a non-invasive assessment of the coronary system [2,3,4]. Stress testing with ECG (“ergometry”) is readily available and relatively inexpensive but has high rates of false positive and false negative results [5]. Stress echocardiography (SE) and myocardial perfusion imaging (MPI) are accepted alternatives that assess coronary myocardial perfusion at rest and under stress (pharmacological or physical) to detect the presence, location, and severity of IHD [3,6].

SE identifies impaired myocardial contractility during conditions of increased oxygen consumption and has a specificity of approximately 87% and a sensitivity of approximately 76% in detecting significant IHD. A limitation of SE is that the patient must meet a heart rate goal in order for the results to be considered clinically relevant. The principle behind SE requires maximal myocardial oxygen consumption; therefore, clinical guidelines state that the patient must reach at least 85% of the age-predicted maximal heart rate (defined as 220 − age) [7,8]. If the target heart rate is not achieved, the patient is often referred for additional imaging tests, such as echo-dobutamine, MPI, or cardiac CT [9,10,11].

We predicted that there would not be a significant benefit in referring patients that are unable to reach the SE heart rate requirement for additional testing for cardiac ischemia. We propose that because the degree of exertion during a sub-maximal stress test is usually higher than the exertion level at which the patients experience chest pain, it still may be efficacious in this subset of patients. We are not aware of previous studies that examined the efficacy of additional testing to detect IHD in patients who do not reach the target heart rate currently required for a stress echo.

The aim of the study was to examine the clinical value of additional imaging tests (cardiac CT, MPI, and/or invasive coronary angiography) for patients that present with chest pain and, despite not reaching their target heart rate, have a normal SE.

## 2. Methods

### 2.1. Study Population and Design

The study population consisted of all consecutive patients, aged ≥18 years, who underwent a stress echo test performed according to the Bruce protocol to assess chest pain at the cardiology department of Soroka University Medical Center (SUMC) between May 2017 and April 2019 and had a normal echocardiographic result. The exclusion criteria consisted of patients with an abnormal SE result, poor imaging of the left ventricle wall, and cardiac disorders, such as significant valvular disease, left bundle branch block (LBBB), hypertrophic cardiomyopathy (HCM), and/or a permanent pacemaker, which reduce the predictive power of SE. We also excluded patients whose test lasted for less than a minute, patients with missing data, and patients who were not members of the health maintenance organization (HMO) affiliated with SUMC.

The study group included all 415 patients who did not reach the target heart rate (calculated by the equation (220 − age) × 85%) and did not show echocardiographic signs of myocardial ischemia. The control group included the first 415 consecutive patients who reached the target heart rate during the stress echo and showed no signs of myocardial ischemia. Since the time period of testing the participants in the control group was limited to the second half of 2017, we verified that the policy of evaluating patients with chest pain did not change during the 2 years of the study.

### 2.2. Clinical Information

The data were derived from the databases of SUMC and the affiliated HMO, Clalit Health Services (CHS). Each individual patient’s chart was carefully reviewed to ensure all clinical and imaging parameters were present at baseline and throughout a 1-year follow-up period. The data parameters included: demographics (age and sex), chronic diseases (hypertension, diabetes, CAD, chronic lung disease, and renal disease), echocardiographic findings at rest and during exertion, results of additional imaging tests (cardiac CT, MPI, and angiography), and any adverse events of myocardial infarction, coronary angioplasty, or bypass surgery, as well as cardiac and overall mortality during the 1-year follow-up period.

### 2.3. Test Information

#### 2.3.1. Stress Echocardiography

Ambulatory or hospitalized patients that met the inclusion criteria were referred to the study by their primary care physician or cardiologist. A symptom-limited graded maximum treadmill exercise test was performed using the Bruce protocol. A 12-lead ECG was continuously monitored and blood pressure was measured at rest and every 3 minutes during the exercise. If patients were on beta blockers, they were asked to stop their medication 24–48 h before the test. If a patient did not stop beta-blocker therapy, it was recorded. Additional medications were instructed to be taken normally prior to the test. During the test, the maximum workload (Mets) was recorded. We used commercially available ultrasound machines to record doppler-echocardiographic images at rest and at peak exercise according to the protocol recommended by the European Association of Echocardiography [7] and the American Society of Echocardiography [12]. Echocardiographic images was taken from parasternal long- and short-axis views and apical four- and two-chamber views using conventional two-dimensional echocardiography. A visual assessment of the endocardial excursion and wall thickening was used for the analysis of stress echocardiograms using a 16-segment model of the left ventricle. The function in each segment was graded at rest and with stress as normal, hyperdynamic, hypokinetic, akinetic, dyskinetic, or aneurysmal. Abnormal study findings included those with fixed wall-motion abnormalities or new or worsening abnormalities indicative of ischemia. In addition to the evaluation of segmental function, the global LV response to stress was assessed. The diastolic function was evaluated in specific clinical conditions.

An abnormal clinical response was defined as any complaint of chest pain (and/or its equivalent) or dyspnea. An abnormal electrocardiographic result was defined as a horizontal or downsloping ST depression of at least 1 mm measured 0.08 s after the J point. An abnormal stress echocardiographic result was defined as a new wall motion abnormality of at least two adjacent myocardial segments of the left ventricular wall during exercise.

#### 2.3.2. Noninvasive Imaging

We included all the available noninvasive imaging tests that were subsequently recorded in the patient’s medical record, including myocardial perfusion imaging (MPI) and/or coronary computed tomography angiography (CCTA). Most tests were performed and reported according to the protocols at the Cardiology Department of Soroka Medical Center. The minority of studies performed outside SUMC were also recorded.

An abnormal MPI examination was defined as a reversible filling defect with an ischemic total perfusion deficit > 3%. An abnormal CCTA scan was defined as a stenosis ≥ 50% in any vessel with a diameter > 2 mm.

#### 2.3.3. Invasive Imaging

Patients were referred for an invasive coronary angiography (ICA) after either an abnormal imaging test or directly at the discretion of their cardiologist or during hospitalizations. An abnormal coronary angiography result was defined as narrowing of ≥50% of the left main coronary artery and ≥ 70% in any coronary segment and/or a positive invasive fractional flow reserve (FFR).

### 2.4. Adverse Cardiac Event

An adverse cardiac event was defined in this study as hospitalization due to acute coronary syndrome (ACS) that required any invasive intervention (percutaneous stent implantation (PCI) or coronary artery bypass grafting (CABG)) and/or resulted in death within 1 year of the stress echo.

### 2.5. Ethics Approval

The study was approved by the institutional review board of the Soroka University Medical Center (Protocol code 0113-18-SOR, approved on 17 April 2018).

### 2.6. Statistical Analysis

The baseline characteristics of the study population were summarized using descriptive statistics. Chi-square tests were used for categorical variables and Fisher’s exact test was used when needed. Continuous variables were compared using a t-test for normally distributed variables and a Mann–Whitney U test for non-normally distributed variables. Results are presented as mean ± standard deviation (SD) for normally distributed continuous variables and as median for non-normally distributed variables. Nominal data are presented as percentages. The level of significance for the examination of the various surgeries was 5% (in two-tailed tests).

To account for the differences in risk factors and reduce selection and statistical bias, we performed a one-to-one exact matching analysis after the data-gathering process. We matched the cases to controls based on age +/− 5 years, hypertension, diabetes, and CAD.

All research data were entered and processed using IBM SPSS Statistics version 26 and figures were created using EdrawMax version 10.0.6.

## 3. Results

### 3.1. Study Population

**Figure 1:**
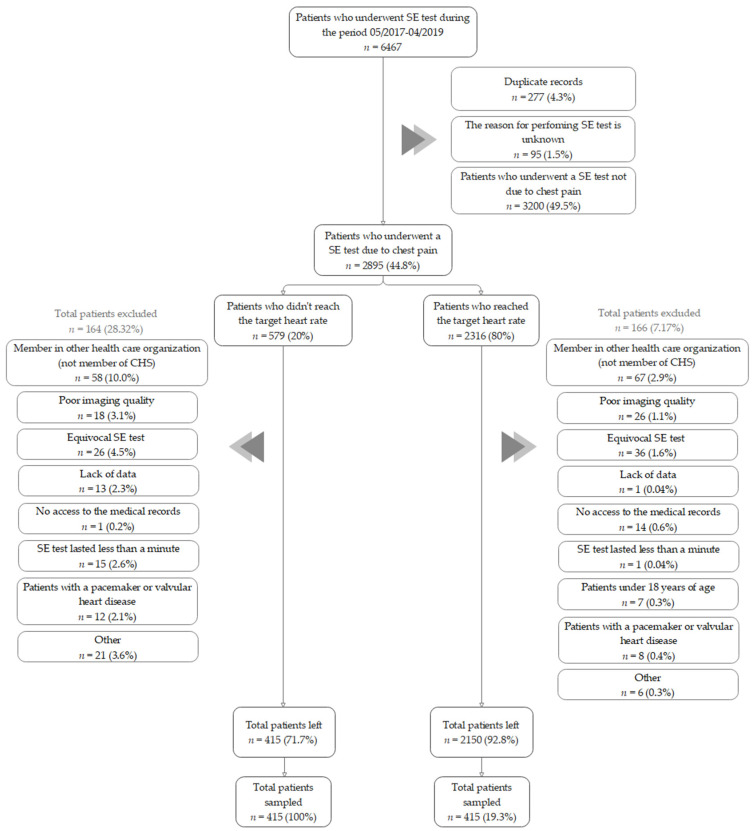
Patient selection flowchart. A total of 6467 SE tests were conducted over a 2-year period. After the initial screening, 3572 patients were excluded. Of the remaining 2895 patients, 330 were further excluded based on the exclusion criteria and 830 patients (415 in each group) were included.

Among the 2895 patients who underwent a stress echo test due to chest pain (during hospitalization or ambulatory) during the study period, 330 patients were excluded due to exclusion criteria or missing follow-up data (Figure 1). The excluded patients were slightly younger but otherwise not significantly different from the included patients (Supplemental Table S1). The final study population included 830 consecutive patients who had a complete follow-up for imaging and adverse cardiac events within a period of 12 months from the day of the test.

### 3.2. Baseline Characteristics

The baseline characteristics of the patient population (mean age 57.5 ± 12.8 years; 54.2% men) stratified by stress echo results are presented in Table 1. Patients who did not reach their target heart rate (the “study group”) were older, more frequently smokers, and had more risk factors and chronic diseases, such as hypertension, diabetes, chronic lung disease, CAD, and/or left ventricular dysfunction. Almost 10% of the patients in the study group took a beta blocker on the day of examination compared to 2.4% in the control group. After matching for age, hypertension, diabetes, and CAD, all the differences between the groups disappeared except for a higher prevalence of left ventricular dysfunction (*p* = 0.03) and the use of beta blockers (*p* ≤ 0.001).

The mean resting heart rate in the study group was lower than in the control group. In order to determine if the difference in mean heart rate was related to taking beta blockers, we compared patients with and without beta-blocker treatment on the day of the test. The mean resting heart rate in the study group was lower than in the control group, regardless of beta-blocker use.

Table 2 and Table 3 show the clinical and electrocardiographic parameters during peak exercise in the two groups. A higher number of subjects in the control group exhibited an abnormal ECG response to stress, possibly because they reached a higher heart rate. In contrast, a clinical response was observed more frequently in the study group, with 23 subjects (5.5%) stopping the exercise because of symptoms, such as chest pain and dyspnea most commonly. The differences between the groups persisted after matching for age, hypertension, diabetes, and CAD.

### 3.3. Downstream Testing

**Figure 2:**
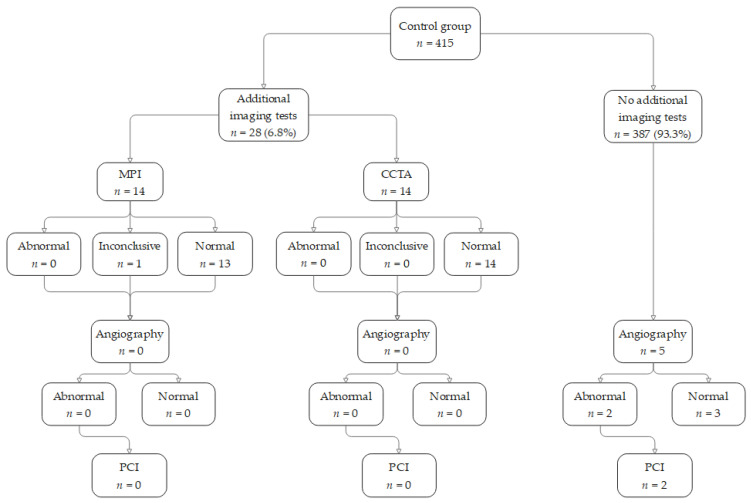
Additional imaging tests that were performed during the follow-up year—control group. Of the 415 patients in the control group, 28 (6.75%) were referred for additional imaging tests, none of which were considered to be abnormal. Two patients finally had a PCI without undergoing additional imaging tests.

**Figure 3:**
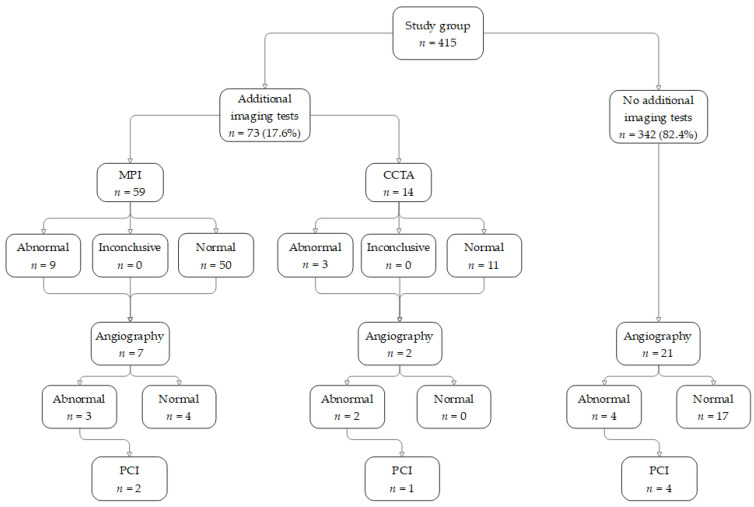
Additional imaging tests that were performed during the follow-up year—study group. Of the 415 patients in the control group, 73 (17.6%) were referred for additional imaging tests, of which 12 were considered to be abnormal. Three of these patients finally had a PCI, with an additional four patients also undergoing PCI without undergoing additional imaging tests.

Table 4 and Figure 2 and Figure 3 show the proportion of patients referred for additional downstream imaging in each of the groups during the 12 months following the stress test. Additional testing was conducted in 73 (17.6%) patients in the study group compared to 28 (6.7%) patients in the control group. In the control group, nine patients were referred by their family physician for a perfusion scan because of ongoing chest pain and/or effort dyspnea, and one for a pre-surgical evaluation. Eight patients were referred by their cardiologist for a perfusion scan and/or cardiac CTA because of ongoing chest pain and/or effort dyspnea. The remaining 10 patients were referred at the request of the hospital physicians during hospitalization for the evaluation of acute chest pain. The median interval between the SE and the additional imaging studies was 84 ± 102 days. Abnormal results were 12 (16.4%) and 0, respectively. Thirty patients in the study group (7.2%) were referred for ICA, although only six were referred after an abnormal imaging test. Nine patients (2.2%) were found to have significant stenosis that required an invasive intervention with PCI: seven of those patients underwent PCI and two patients did not undergo PCI for reasons of non-cooperation, and eventually were treated conservatively. Five of the seven patients who underwent PCI underwent the SE during hospitalization for chest pain, while the other two were referred for an ambulatory SE.

In the control group, 28 patients underwent an additional imaging test, and none showed abnormal results. Five patients were referred for an angiography (without a preceding imaging test), and, of these, two underwent a PCI. The difference in PCI performance between the groups was not statistically significant (*p* = 0.2), and both rates lie within the accepted false negative rates of a stress echocardiography. Overall, 26 patients from both groups were referred for an angiography without additional imaging studies. The majority had additional risk factors and were referred during hospitalization for chest pain, unstable angina, or acute MI. Five of these patients underwent a PCI, while the remaining were found to have angiographically normal arteries or non-obstructive coronary disease.

Table 5 shows that the majority of the patients who underwent a PCI had one or more of the following: diabetes, hypertension, documented CAD, and/or use of beta blockers. None of these patients reached a heart rate above 78% of their target heat rates.

### 3.4. Adverse Cardiac Event

Of the nine patients in the study group who met the definition of an adverse cardiac event, four were hospitalized with ACS (one with unstable angina, three with NSTEMI), and all underwent a PCI during the same hospitalization. Two other patients were referred electively for a coronary angiography after an abnormal imaging test (CCTA\MPI). One patient was hospitalized with chronic stable angina and underwent a PCI during the same hospitalization. The two remaining patients were found to have significant stenosis in the ICA but were treated conservatively due to a lack of cooperation. In the control group,10 patients were hospitalized for acute chest pain but only two were diagnosed with ACS (one with unstable angina, the other with STEM) and underwent a PCI.

## 4. Discussion

In this study, we examined patterns of downstream testing and outcomes in a cohort of 830 patients referred for stress echocardiography. In 82.4% of the subjects in the study group no further imaging was performed during the follow-up period. Of the 73 patients of the study group who did undergo additional imaging studies (MPI or CCTA), only 12 patients had an abnormal result, of whom nine underwent an invasive angiography and three required a PCI. Of those that did not undergo additional imaging studies, 21 were referred for an angiography during a subsequent hospitalization for chest pain, ACS, or for the evaluation of left ventricular dysfunction, but only four were found to be abnormal and required a PCI. Overall, only a minority (2.2%) of all the subjects in the study group had a coronary event and/or needed invasive coronary intervention during a period of 1 year since the day of the test. No patient underwent a CABG and there were no deaths in this study. This proportion of patients that experienced a coronary event lies within the range of expected false negative tests of the SE, even in subjects who achieve their target heart rate. This finding supports our main research hypothesis: in patients experiencing chest pain during daily activities that do not show wall motion abnormalities at peak stress, there is a low probability of significant myocardial ischemia, even when the patient’s peak stress is not accompanied by meeting the target heart rate. Our study also supports the notion that normal stress echocardiography results predict low rates of CAD events in patients with acute, as well as stable, chest pain [3]. Since the follow-up of our study group was only 1 year, we suggest that the warranty period of SE within individual exercise tolerance should be limited to 1 year.

Of the patients who were indicated for revascularization but were not detected in the stress echo test due to a failure to achieve the target heart rate, eight out of nine of these patients had at least one of the following medical conditions: hypertension, diabetes, and/or known ischemic heart disease. This is in accordance with the study by Niloufar Samiei et al. [13], who found that in patients with a negative stress test result or from dobutamine echocardiographic studies MACE occurred more frequently among older (≥65 years) men with preexisting diabetes, hypertension, and/or hyperlipidemia. Most of these patients underwent the SE during hospitalization for chest pain. Five of these patients took a beta blocker within 24 h prior to the test, and none reached a heart rate above 78% of their target heart rate. Thus, we suggest that patients who are less likely to benefit from an additional imaging test are those without a known risk factor, who discontinue beta-blocker administration 24–48 h prior to the SE, and who are able to achieve a heart rate of at least 78% of their target heart rate according to current guidelines.

In a review of the literature, we did not find a study that specifically examined our research question—the value of follow-up tests after an inconclusive stress echo test. Bitencourt and colleagues did examine the prevalence and value of additional downstream testing in patients who underwent an exercise treadmill test (ETT) [14]. They divided their patients into three subgroups—a negative, indecisive, or positive stress test result. The definition of an indecisive test included the inability to reach the target heart rate, an abnormal baseline ECG, an abnormal stress ECG that returned to normal in less than a minute, patients with typical angina, and/or shortness of breath without an abnormal stress ECG. They found that patients who benefited least from follow-up tests were those who showed a rapid recovery of the ECG changes or a negative ETT, and those patients who benefited most were those with typical angina despite a negative ETT and/or patients with a positive ETT. The results of our study indicate that clinical symptoms without echocardiographic ischemic changes were not indicative of an adverse cardiac event.

An expected yet important finding that emerged from our study is that beta blockers negatively affect the efficacy of a stress echocardiography test in detecting ischemic heart disease. The proportion of patients taking beta blockers in the study group (most of them for the treatment of hypertension) was about four times greater than in the control group (9.9 vs. 2.4%), and the mean maximal heart rate reached by these patients was lower than the mean heart rate of those who did not take beta blockers. Indeed, five of the nine patients with an adverse cardiac event (55%) took a beta blocker on the day of the test. Because of the retrospective nature of our study, it is not possible to determine whether beta blockers made it difficult for these patients to reach the required heart rate or whether patients who needed beta blockers were already in a more complex health condition and were more likely to have ischemic heart disease. We suggest that stress echo studies should ideally be avoided within 24–48 h of taking a beta blocker.

### Study Limitations and Strengths

This study was conducted at a single medical center, which may not represent the entire population nationally or globally. The number of participants in the study and the number of adverse cardiac events may be too small to detect statistically significant differences in some of the variables. The fact that we recruited only the first 415 consecutive patients of the 2150 eligible patients in the control group may have introduced some bias to patient selection, although we believe that choosing consecutive patients attenuated this possibility. Due to a lack of data, we did not examine the effect of other risk factors, such as obesity, dyslipidemia, a history of premature heart disease in the family, etc. Thus, we recommend performing a multicenter prospective study involving a larger number of subjects to examine the clinical value of additional imaging after a negative stress echo.

The major strength of our study was the analysis of the individual patient charts, which are incredibly comprehensive due to the nature of the health care system. Almost all inpatient and outpatient imaging studies of CHS patients in the southern district of Israel are performed at SUMC because it is the sole tertiary medical center in southern Israel.

## 5. Conclusions

Our study suggests that a target heart rate of 85% for stress echocardiography may be too ambitious, and that in most patients with chest pain who do not show ischemic changes on a stress echocardiogram additional imaging studies can be safely deferred, as long as a heart rate above 78% of the maximal calculated heart rate is reached. Specific caution should be taken in those that have underlying risk factors (diabetes, hypertension, and/or known IHD), and especially in those who take a beta blocker within 24 h of the test, for whom additional imaging studies should be considered.

## Figures and Tables

**Table 1:** Baseline demographic and clinical characteristics of the study population.

	Study (*n* = 415)	Control (*n* = 415)	*p*-Value
Male, no. (%)	225 (54.2)	223 (53.7)	0.9
Age (years), mean ± SD	58.6 ± 13.1	55.9 ± 12.3	0.003
Hypertension no. (%)	210 (50.6)	153 (36.9)	<0.001
Diabetes no. (%)	132 (31.8)	95 (22.9)	0.004
Lung disease no. (%)	45 (10.8)	29 (7)	0.05
Kidney disease no. (%)	23 (5.5)	16 (3.9)	0.3
CAD no. (%)	106 (25.5)	43 (10.4)	<0.001
Current smoker no. (%)	143 (34.5)	103 (24.8)	<0.001
Abnormal ECHO no. (%)	39 (9.4)	25 (6)	0.07
Beta blockers no. (%)	41 (9.9)	10 (2.4)	<0.001
Heart rate at rest, mean ± SD. median	78.9 ± 14.9, 77	83.4 ± 13.7, 82	<0.001
No BB ^a^	79.9 ± 15.1, 78	83.3 ± 13.8, 82	<0.001
On BB ^b^	70.5 ± 9.7, 69	86.3 ± 8.8, 89	<0.001
Systolic BP at rest	127.4 ± 13.8	125.9 ± 11.2	0.09
No BB	127.4 ± 13.6	125.9 ± 11.2	0.08
On BB	127.3 ± 15.8	128 ± 10.3	0.9
Diastolic BP at rest	78.8 ± 5.8	78.9 ± 6.3	0.7
No BB	78.9 ± 5.8	78.9 ± 6.3	0.8
On BB	77.8 ± 6.1	77 ± 6.7	0.7

Abbreviations: BP, blood pressure; BB, beta blockers. ^a^ No BB, patients who stopped taking beta blockers within 24 h prior to the day of the stress echo test. ^b^ On BB-patients who had not stopped taking beta blockers 24 h prior to the day of the stress echo test.

**Table 2:** Parameters at peak exercise.

	Study (*n* = 415)	Control (*n* = 415)	*p*-Value
Heart rate, mean ± SD. median	121.7 ± 15.6, 123	149.9 ± 11.8, 150	<0.001
No BB	122.6 ± 15.6, 123	149.9 ± 11.8, 150	<0.001
On BB	114.1 ± 13.9, 115	151.5 ± 10.5, 148	<0.001
Systolic BP	145.7 ± 20.9	154.4 ± 15.1	<0.001
No BB	145.8 ± 21.2	154.3 ± 15.0	<0.001
On BB	145.1 ± 18.3	156 ± 18.4	0.1
Diastolic BP	80.7 ± 10.2	80.3 ± 6.5	0.5
No BB	80.9 ± 10.5	80.3 ± 6.6	0.3
On BB	79.0 ± 6.2	80 ± 0	0.6
MPHR (%), mean ± SD	75.2 ± 7.3	91.2 ± 4.1	<0.001
No BB	75.5 ± 7.3	91.2 ± 4.1	<0.001
On BB	72.6 ± 6.7	91 ± 4.9	<0.001
METs	7.9 ± 2.7	9.1 ± 2.6	<0.001
Duration (minutes)	5:34 ± 2:23	6:20 ± 1:57	<0.001

Abbreviations: MPHR, maximal predicted heart rate; METs, metabolic equivalents.

**Table 3:** Symptoms and ECG at peak exercise.

	Study (*n* = 415)	Control (*n* = 415)	*p*-Value
ECG at peak exercise, no. (%)			<0.001
Abnormal ECG ^a^	39 (9.4)	82 (19.8)	
Uncertain	2 (0.5)	1 (0.2)	
Clinical response			<0.001
Yes	43 (10.4)	4 (1)	
Uncertain response	35 (8.4)	5 (1.2)	
Description			<0.001
Chest pain	18 (4.3)	6 (1.4)	
Dyspnea	27 (6.5)	1 (0.2)	
Other ^b^	30 (7.2)	1 (0.2)	

^a^ Abnormal ECG defined as horizontal/downsloping ST depression of at least 1 mm or ST elevation for 60–80 msec after QRS. ^b^ Other defined as tiredness or lightheadedness.

**Table 4:** Downstream testing and results.

	Study (*n* = 415)	Control (*n* = 415)	*p*-Value
MPI, no. (%)	59 (14.2)	14 (3.4)	<0.001
Abnormal	9 (2.2)	0 (0)	
Normal	50 (12.0)	13 (3.1)	
Inconclusive	0 (0)	1 (0.2)	
CCTA	14 (3.3)	14 (3.3)	0.3
Abnormal	3 (0.7)	0 (0)	
Normal	11 (2.6)	14 (3.3)	
Angiography	30 (7.2)	5 (1.2)	<0.001
Abnormal	9 (2.1)	2 (0.5)	
Normal	21 (5.1)	3 (0.7)	
PCI	7 (1.7)	2 (0.5)	0.2

Abbreviations: MPI, myocardial perfusion imaging; CCTA, coronary computed tomography angiography; PCI, percutaneous coronary intervention.

**Table 5:** Comparison between patients with adverse cardiac events in the study group and those without.

	Patients with Adverse Cardiac Events (*n* = 9)	Patients without Adverse Cardiac Events (*n* = 406)	*p*-Value
Male, no. (%)	6 (66.7)	219 (53.9)	0.5
Age (years), mean ± SD	584 ± 6.8	58.6 ± 13.3	0.7
Chronic diseases	8 (88)	276 (68)	
≥2 chronic diseases	5 (55.6)	150 (36.9)	0.4
hypertension	7 (77.8)	203 (50)	0.2
known CAD	4 (44.4)	102 (25.1)	0.2
Diabetes	4 (44.4)	128 (31.5)	0.5
Lung disease	0	45 (11.1)	0.6
Kidney disease	0	23 (5.7)	1
METs achieved	8.4 ± 3.7	7.9 ± 2.6	1
Target heart rate achieved %			0.03
<70	1 (11.1)	76 (18.7)	
70–75	3 (33.3)	92 (22.7)	
76–80	5 (55.6)	121 (29.8)	
>80	0	117 (28.8)	
On BB	4 (44.4)	37 (9.1)	0.007
Abnormal ECHO	0	39 (9.6)	1
Clinical response			0.7
Yes	1 (11.1)	42 (10.3)	
Uncertain response	1 (11.1)	34 (8.4)	
Abnormal ECG at peak exercise	3 (33.3)	36 (8.9)	0.002

## Supplementary Materials

The following supporting information can be downloaded at: https://www.mdpi.com/article/10.3390/jcm12144832/s1, Table S1. Comparison of included and excluded subjects.
