# Supplementary material for: Downstream Imaging Studies Do Not Significantly Improve Outcome in Most Patients with Chest Pain Who Did Not Reach Their Target Heart Rate on a Stress ECHO Study
Source: J Clin Med. 2023 Jul 22;12(14):4832. doi: 10.3390/jcm12144832 (PMC10381276; doi:10.3390/jcm12144832)
Supplement: Supplementary file 1 [file jcm-12-04832-s001.zip › jcm-2445892-supplementary.pdf]

Supplementary Material: Downstream Imaging Studies Do Not Significantly Improve Outcome in Most Patients with Chest Pain Who Did Not Reach Their Target Heart Rate on a Stress ECHO Study

Nativ Henkin, Ifat Karilker, Sergio L. Kobal, Rachel Golan, Aryeh Shalev, Shaul Atar and Yaakov Henkin

Table S1. Comparison of included and excluded subjects.

	**Included (n = 830)**	**Excluded (n = 330)**	***p*-Value**
Male, No. (%)	225 (54.2)	223 (53.7)	0.9
Age (years), mean ± SD	58.6 ± 13.1	55.9 ± 12.3	0.003
Abnormal ECHO	39 (9.4)	25 (6)	0.07
Parameters at rest			
Heart rate, mean ± SD. median	81.0 ± 14.9, 80	81.9 ± 13.5, 81	0.2
Systolic BP	126.8 ± 13.5	128 ± 14.3	0.07
Diastolic BP	78.8 ± 6.1	79.3 ± 7.7	0.9
Parameters at peak exercise			
Heart rate, mean ± SD. median	135.8 ± 19.7, 137	137.4 ± 22.2, 141	0.1
Systolic BP at peak exercise	150.1 ± 18.7	152.8 ± 21.6	0.06
Diastolic BP at peak exercise	80.5 ± 8.5	80.1 ± 9.9	0.5
MET's	8.7 ± 4.8	8.8 ± 3.3	0.7
